# Secular trend analysis of lung cancer incidence in Sihui city, China between 1987 and 2011

**DOI:** 10.1186/s40880-015-0037-3

**Published:** 2015-07-31

**Authors:** Jin-Lin Du, Xiao Lin, Li-Fang Zhang, Yan-Hua Li, Shang-Hang Xie, Meng-Jie Yang, Jie Guo, Er-Hong Lin, Qing Liu, Ming-Huang Hong, Qi-Hong Huang, Zheng-Er Liao, Su-Mei Cao

**Affiliations:** Sun Yat-sen University Cancer Center, State Key Laboratory of Oncology in South China, Collaborative Innovation Center for Cancer Medicine, Guangzhou, Guangdong 510060 P. R. China; Department of Cancer Prevention Research, Sun Yat-sen University Cancer Center, Guangzhou, Guangdong 510060 P. R. China; School of Public Health, Guangdong Medical University, Dongguan, Guangdong 523808 P. R. China; Sihui Cancer Institute, Sihui, Guangdong 526200 P. R. China; Department of Clinical Trial Center, Sun Yat-sen University Cancer Center, Guangzhou, Guangdong 510060 P. R. China; Department of Cancer Screening, Sun Yat-sen University Cancer Center, Guangzhou, Guangdong 510060 P. R. China

**Keywords:** Lung cancer, Secular trend, Joinpoint regression analysis, Age–period–cohort model

## Abstract

**Background:**

With industrial and econom ic development in recent decades in South China, cancer incidence may have changed due to the changing lifestyle and environment. However, the trends of lung cancer and the roles of smoking and other environmental risk factors in the development of lung cancer in rural areas of South China remain unclear. The purpose of this study was to explore the lung cancer incidence trends and the possible causes of these trends.

**Methods:**

Joinpoint regression analysis and the age–period–cohort (APC) model were used to analyze the lung cancer incidence trends in Sihui, Guangdong province, China between 1987 and 2011, and explore the possible causes of these trends.

**Results:**

A total of 2,397 lung cancer patients were involved in this study. A 3-fold increase in the incidence of lung cancer in both sexes was observed over the 25-year period. Joinpoint regression analysis showed that while the incidence continued to increase steadily in females during the entire period, a sharp acceleration was observed in males starting in 2005. The full APC model was selected to describe age, period, and birth cohort effects on lung cancer incidence trends in Sihui. The age cohorts in both sexes showed a continuously significant increase in the relative risk (RR) of lung cancer, with a peak in the eldest age group (80–84 years). The RR of lung cancer showed a fluctuating curve in both sexes. The birth cohorts identified an increased trend in both males and females; however, males had a plateau in the youngest cohorts who were born during 1955–1969.

**Conclusions:**

Increasing trends of the incidence of lung cancer in Sihui were dominated by the effects of age and birth cohorts. Social aging, smoking, and environmental changes may play important roles in such trends.

## Background

Lung cancer is the leading cause of cancer-related mortality worldwide, with 1.82 million new cases and 1.2 million deaths per year [1, 2]. The high-risk areas for lung cancer include central-eastern and southern Europe, North America, and eastern Asia [3]. In 1980, it was estimated that 31% of lung cancer cases occurred in developing countries; in 2012, however, the majority of lung cancer cases (55%) occurred in developing countries [4]. Males have a higher incidence of lung cancer than females, with a sex ratio of 2.5:1 [2, 3]. In China, lung cancer is also the most common and deadly cancer in males [4, 5], with an incidence of 70.39/100,000 and a mortality of 56.72/100,000 in 2010 [6]. For females, the incidence and mortality were 39.47/100,000 and 27.04/100,000, respectively, in 2010 [6].

Sihui city is located in the midwestern Guangdong province, with an area of 1,163 square kilometers. In 2011, the population of Sihui was 418,097 and >75% lived in rural residences. With the development of industry and the growth of the economy in the last 30 years, the lifestyle and living conditions have changed significantly in China. Because lung cancer is mainly caused by cigarette smoking and environmental factors, analysis of the long-term incidence of lung cancer in Sihui will provide valuable information for illustrating the roles of lifestyle and environment in the development of lung cancer in rural areas of South China.

In the current study, we used joinpoint regression and age–period–cohort (APC) analyses with a Poisson regression model to determine the secular trends in the incidence of lung cancer in Sihui between 1987 and 2011.

## Methods

### Data sources

The data involving all incident lung cancer cases diagnosed between January 1, 1987 and December 31, 2011 (Table 1) were obtained from the Sihui Cancer Registry and analyzed by using year of diagnosis, age at diagnosis, gender, and histologic codes. The International Classification of Diseases (ICD) code corresponding to lung cancer was 162 in the 9th revision (1987–1994), and C33 and C34 in the 10th revision (1995–2004). Populations were estimated on July 1 of each year based on the Chinese population census reports of 1990, 2000, and 2010.

**Table 1:** The distributions of lung cancer cases and populations by age and sex in the period of 1987–2011 in Sihui city, Guangdong province, China

Age group	Cases^a^					Populations (×1,000)^a^				
	1987–1991	1992–1996	1997–2001	2002–2006	2007–2011	1987–1991	1992–1996	1997–2001	2002–2006	2007–2011
10–14	0/0	0/0	0/0	2/0	0/0	80/76	77/68	87/78	92/81	90/78
15–19	0/0	0/0	0/0	0/1	0/1	99/95	79/75	93/84	90/82	90/83
20–24	0/0	2/0	0/0	2/0	0/0	97/95	98/95	91/88	79/74	81/76
25–29	4/0	3/2	3/1	0/1	3/0	69/66	97/92	93/86	99/90	99/91
30–34	3/0	4/2	1/2	4/1	2/2	69/66	66/63	85/80	105/97	101/95
35–39	2/4	4/3	5/3	11/9	6/5	64/58	70/66	79/74	91/84	93/88
40–44	12/2	12/4	10/3	17/4	17/14	46/38	61/55	57/53	63/61	71/70
45–49	8/3	16/7	18/7	25/10	30/10	38/33	42/36	50/46	63/60	68/66
50–54	20/7	23/5	23/7	36/9	52/19	37/37	37/32	42/39	48/44	51/49
55–59	18/7	20/13	40/10	43/14	76/34	31/37	35/37	34/35	35/31	40/38
60–64	31/10	60/15	50/18	59/15	83/18	28/33	32/35	39/33	31/30	34/34
65–69	25/7	43/20	49/16	60/19	100/29	23/27	25/31	24/30	26/32	28/32
70–74	27/6	50/16	52/14	63/26	99/50	16/23	19/25	16/25	20/28	21/28
75–79	13/3	22/13	22/7	44/12	84/41	10/18	13/19	10/18	13/20	14/22
80–84	2/2	14/13	13/2	11/5	43/32	2/14	7/14	4/12	5/13	6/14
85	1/0	4/3	9/4	4/9	17/15	1/14	5/18	1/8	2/10	3/11

^a^All data are presented as the numbers of males/females.

### Statistical methods

Age-standardized rates (ASRs) of incidence over 25 years were calculated with the direct method according to the Segi’s world standard population in 1962, and the secular trends in lung cancer were compared between both sexes. Long-term trends of ASRs of incidence were analyzed by using a joinpoint regression model, which was developed by the United States National Cancer Institute for the analysis of data from the Surveillance Epidemiology and End Results Program [7]. Joinpoint regression analysis describes changes in data trends by connecting several different line segments on a log scale at joinpoints [8]. The analyses in this study started with the minimum number of joinpoints and tested for model fit with a maximum of four joinpoints. An annual percent change in the ASR of incidence for each line segment and the corresponding 95% confidence interval (CI) was estimated. The Monte Carlo permutation method was used to test for statistical significance [8–10].

A Poisson regression analysis was performed for the effects of age, period, and birth cohort on the incidence of lung cancer for each gender. In this analysis, we used the logarithm of the incidence of lung cancer as the dependent variable, and age, period, and birth cohort as independent variables [11–14]. The periods were arranged in five 5-year periods between 1987 and 2011, the ages were stratified by 5-year age groups from 35–39 years to 80–84 years, and 14 corresponding birth cohorts were included from 1905–1909 to 1970–1974 (Table 2). The 45–49 years age group, the 1997–2001 period group, and the 1915–1919 birth cohort group were used as reference groups, and the results of this model were more reliable than those using the groups of the fewer age-specific rates as the reference groups [15–17]. The deviances of different models and the *P* value of the Chi square test of the goodness-of-fit were used to check the model fit. The closer the deviance to the degrees of freedom, the better the model fit; the greater the *P* value of the goodness-of-fit, the better the data fit [15, 16]. Relative risk (RR) of incidences was calculated to summarize the effects of age, period, and birth cohorts from APC models.

**Table 2:** The distributions of birth cohorts based on diagnostic age and period for lung cancer cases in age–period–cohort models

Age group	Period				
	1987–1991	1992–1996	1997–2001	2002–2006	2007–2011
35–39	1950–1954	1955–1959	1960–1964	1965–1969	1970–1974
40–44	1945–1949	1950–1954	1955–1959	1960–1964	1965–1969
45–49	1940–1944	1945–1949	1950–1954	1955–1959	1960–1964
50–54	1935–1939	1940–1944	1945–1949	1950–1954	1955–1959
55–59	1930–1934	1935–1939	1940–1944	1945–1949	1950–1954
60–64	1925–1929	1930–1934	1935–1939	1940–1944	1945–1949
65–69	1920–1924	1925–1929	1930–1934	1935–1939	1940–1944
70–74	1915–1919	1920–1924	1925–1929	1930–1934	1935–1939
75–79	1910–1914	1915–1919	1920–1924	1925–1929	1930–1934
80–84	1905–1909	1910–1914	1915–1919	1920–1924	1925–1929

A Poisson regression model and other statistical analyses were performed by using the SAS statistical software (version 9.2; SAS Institute, Cary, NC, USA). For all analyses, the significance level was set at *P* ≤ 0.05.

## Results

**Fig. 1:**
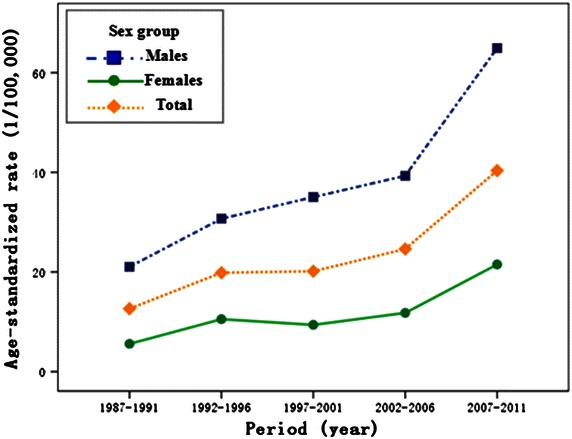
Age-standardized rates (ASRs) of lung cancer incidence by sex during 1987–2011 in Sihui, China.

**Fig. 2:**
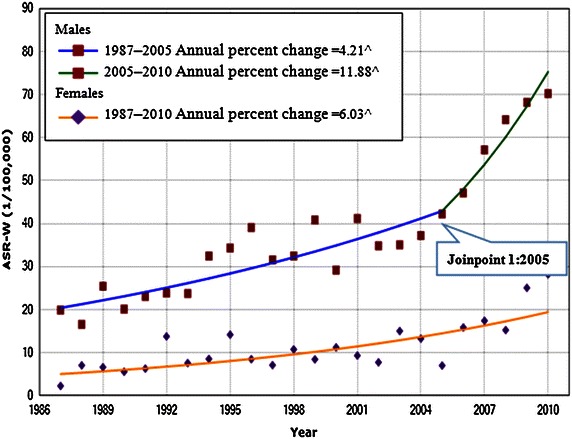
The trends in world age-standardized rates (ASR-W) of lung cancer incidence for both sexes in the period of 1987–2010 in Sihui with joinpoint regression by sex. ^Denotes parameters significantly different from zero at α = 0.05.

Between 1987 and 2011, a total of 2,397 lung cancer patients were diagnosed in Sihui city, with a male-to-female ratio of 2.60:1. The average crude incidences of lung cancer in males, females, and the overall population were 35.20/100,000, 14.18/100,000, and 24.93/100,000 and the corresponding ASRs were 37.27/100,000, 11.52/100,000, and 23.05/100,000, respectively (Table 3). The median age at the time of diagnosis for lung cancer in Sihui was 65 years. The ASRs of lung cancer incidence showed upward trends in both males and females, with a 3-fold increase during the entire period (Fig. 1). In females, there was a steady growth of the annual percent change (6.03%; 95% CI, 3.8%–8.3%), and no joinpoint was included in the joinpoint regression model. In males, the increase in trend accelerated between 2005 and 2010 with an annual percent change of 11.9% (95% CI, 3.1%–21.4%) (Fig. 2).

**Table 3:** Lung cancer incidences in Sihui, Guangdong, China between 1987 and 2011

Period	Males				Females				Overall			
	Population	Cases	CR (1/10^5^)	ASR-W^a^ (1/10^5^)	Population	Cases	CR (1/10^5^)	ASR-W^a^ (1/10^5^)	Population	Cases	CR (1/10^5^)	ASR-W^a^ (1/10^5^)
1987–1991	873,556	166	19.00	21.04	864,750	51	5.90	5.58	1,738,306	217	12.48	12.64
1992–1996	940,056	277	29.47	30.70	902,640	116	12.85	10.53	1,842,696	393	21.33	19.86
1997–2001	1,004,990	295	29.35	35.04	947,545	94	9.92	9.39	1,952,535	389	19.92	20.15
2002–2006	1,040,108	381	36.63	39.31	974,515	135	13.85	11.79	2,014,623	516	25.62	24.62
2007–2011	1,059,544	612	57.76	60.26	1,007,397	270	26.80	20.29	2,066,941	882	42.67	37.98
Average	4,918,254	1,731	35.20	37.27	4,696,847	666	14.18	11.52	9,615,101	2,397	24.93	23.05

*CR* crude rate, *ASR-W* world age-standardized rate.

^a^ASR-W was calculated with the direct method using Segi’s world standard population in 1962.

Poisson regression analysis was performed to observe the effects of age, period, and birth cohort on the incidence of lung cancer in both sexes. Five calendar periods (1987–1991, 1992–1996, 1997–2001, 2002–2006, 2007–2011) and 10 age groups (5-year periods from 35–39 years to 80–84 years) were used in all Poisson regression models; thus, 14 corresponding birth cohorts were included from 1905–1909 to 1970–1974. The full APC model fit the data best in both males and females based on the deviance statistic and was chosen as the final model (Table 4).

**Table 4:** Comparison of age–period–cohort models for lung cancer incidences with Poisson regression in Sihui

Model	DF	Males		Females	
		Dev	*P* value^a^	Dev	*P* value^a^
Age	40	244.5	<0.001	187.3	<0.001
Age–period	36	56.7	0.008	56.6	0.048
Age–cohort	27	42.3	0.047	50.3	0.009
Age–period–cohort	24	29.8	0.252	24.1	0.562
Period curvature effect^b^	3	12.5	0.007	26.2	<0.001
Cohort curvature effect^b^	12	26.9	0.003	32.5	0.002

*DF* degree of freedom, *Dev* deviance.

^a^ *P* value was from a Chi square test of the goodness-of-fit of the model.

^b^Effects were assessed by comparing the age–period–cohort model with the age-cohort model (period curvature effect) and the age–period–cohort model with the age-period model (cohort curvature effect).

**Fig. 3:**
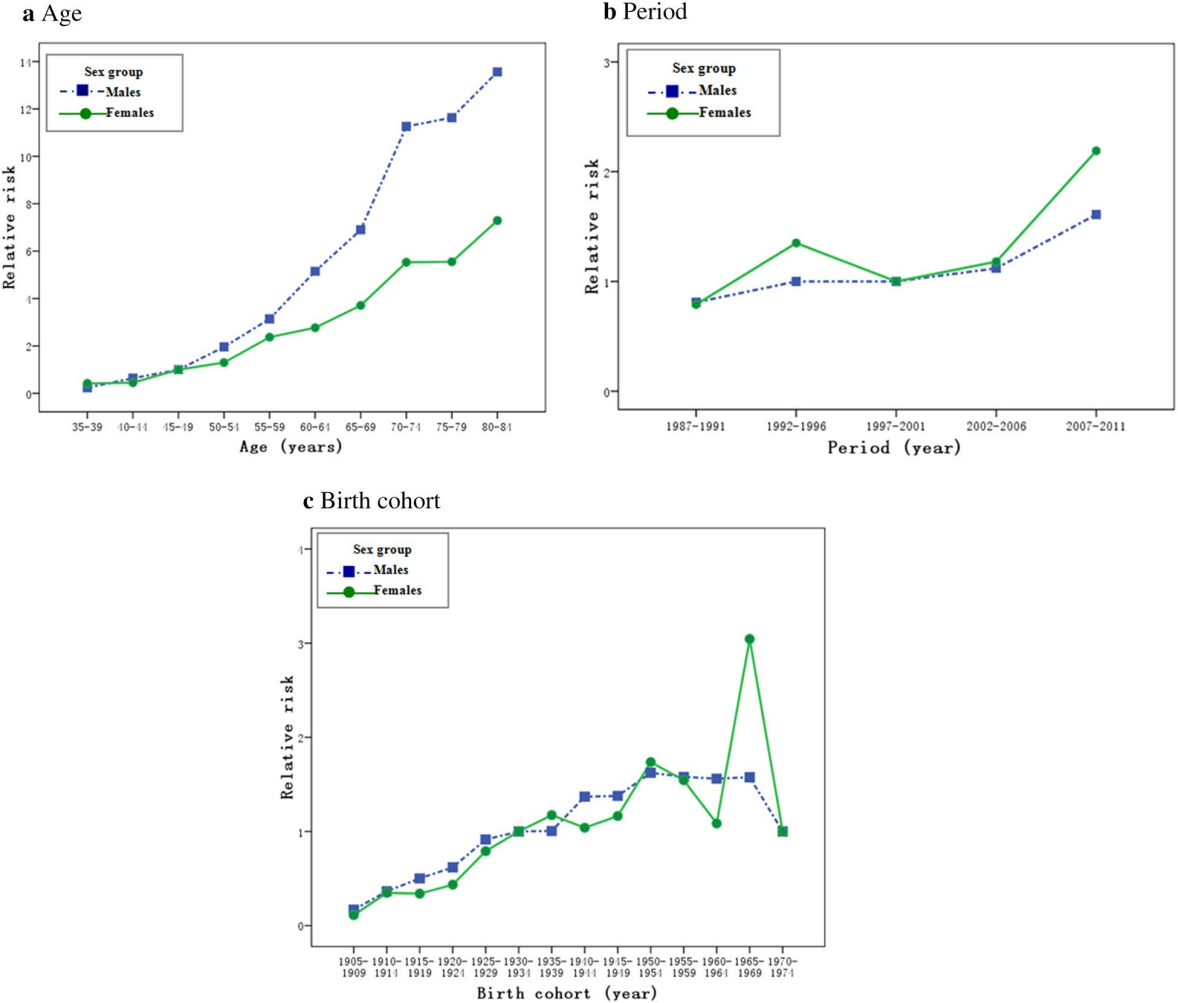
Effect of age, period, and birth cohort on the relative risk of lung cancer incidence with age–period–cohort analysis in Sihui by sex. **a** Age; **b** period; **c** birth cohort.

After adjusting for period and cohort effects, the RR of age on the incidence of lung cancer showed a continuously rising trend until the oldest age cohort (80–84 years) for both sexes (Table 5; Fig. 3a). The RR showed a relatively flat trend, with only a slight increase in the most recent period (2007–2011) in both sexes (Table 5; Fig. 3b). By using the 1915–1919 birth cohort as a reference group, the effects of the birth cohort curve fluctuated, but there was a significant continuous increase in RR during the entire period in females (Table 5; Fig. 3c). The first birth cohort (1905–1909) and the last birth cohort (1970–1974) were not shown because they each contained only one case. The RR continued to increase in males from the 1910–1914 birth cohort to the 1950–1954 birth cohort when it reached its peak; subsequently, there was a plateau in the younger cohort born during 1955–1969.

**Table 5:** The relative risk (RR) estimates with 95% confidence interval (CI) of lung cancer incidence in the age–period–cohort model for both sexes in the period of 1987–2011 in Sihui

Parameter	Subgroup	Males	Females
		RR (95% CI)	RR (95% CI)
Age (years)	35–39	0.24 (0.14, 0.38)^a^	0.42 (0.23, 0.73)^b^
	40–44	0.64 (0.44, 0.94)^a^	0.45 (0.24, 0.80)^b^
	45–49	Reference	Reference
	50–54	1.96 (1.43, 2.72)^b^	1.30 (0.81, 2.11)
	55–59	3.14 (2.21, 4.53)^a^	2.37 (1.47, 3.90)^b^
	60–64	5.15 (3.47, 7.89)^a^	2.77 (1.63, 4.84)^b^
	65–69	6.90 (4.34, 11.48)^a^	3.71 (2.07, 6.88)^a^
	70–74	11.26 (6.63, 20.37)^a^	5.53 (2.93, 10.94)^a^
	75–79	11.63 (6.32, 23.20)^a^	5.55 (2.73, 11.98)^a^
	80–84	13.56 (6.61, 30.46)^a^	7.29 (3.23, 17.59)^a^
Period (year)	1987–1991	0.81 (0.61, 1.09)	0.79 (0.52, 1.18)
	1992–1996	1.00 (0.81, 1.24)	1.35 (0.99, 1.83)
	1997–2001	Reference	Reference
	2002–2006	1.12 (0.92, 1.37)	1.18 (0.88, 1.58)
	2007–2011	1.61 (1.25, 2.03)^a^	2.19 (1.62, 2.96)^a^
Cohort (year)	1910–1914	0.78 (0.45, 1.32)	1.13 (0.56, 2.28)
	1915–1919	Reference	Reference
	1920–1924	1.13 (0.80, 1.61)	1.16 (0.69, 1.99)
	1925–1929	1.56 (1.12, 2.21)^b^	1.92 (1.22, 3.09)^b^
	1930–1934	1.73 (1.20, 2.57)^b^	2.19 (1.38, 3.61)^b^
	1935–1939	1.51 (0.98, 2.44)	2.33 (1.40, 4.05)^b^
	1940–1944	1.90 (1.15, 3.36)^b^	1.87 (1.04, 3.55)^b^
	1945–1949	1.85 (1.04, 3.60)^b^	1.90 (0.98, 3.92)
	1950–1954	2.05 (1.08, 4.37)^b^	2.57 (1.29, 5.62)^b^
	1955–1959	1.87 (0.90, 4.41)	2.07 (0.95, 5.00)
	1960–1964	1.75 (0.76, 4.58)	1.32 (0.52, 3.68)
	1965–1969	1.67 (0.66, 4.91)	3.36 (1.34, 9.71)^b^

^a^ *P* < 0.001.

^b^ *P* < 0.05.

## Discussion

This study showed that the incidence of lung cancer has been rapidly increasing in Sihui over the last 25 years. Although the overall incidence of lung cancer in Sihui (24.93/100,000) was lower than the average incidence of lung cancer in China (46.08/100,000) [6], the rate of increase (5.90%) was greater than the average in China (1.64%) [18]. The APC analysis further revealed that the increasing trend in the incidence of lung cancer was mainly due to the effects of aging population and young birth cohort in both sexes. Such temporal patterns may indicate the complex impact of lifestyle and environment changes on the risk of lung cancer in rural areas of South China [5, 19–22].

Sihui is a county-level city located in the central part of Guangdong province. Before the era of economic reformation and open policy in China, Sihui was a traditional agricultural location with rice paddies and oranges as the main economic crops. Corresponding with the implementation of a policy of openness and reform by Chinese government in 1978, Sihui became one of the earliest economic boom areas in South China. The major industries in Sihui include jade processing, smelting, metallurgic casting, and ceramic manufacturing. As a result of the industrial needs, most of the laborers, especially males, moved from agricultural areas to local factories and therefore a series of life style and environmental changes occurred.

It has been estimated that cigarette smoking causes >75% of all lung cancers [23]. Other known and suspected risk factors for lung cancer include a history of tuberculosis and other inflammatory lung diseases [24, 25], indoor air pollution [26–28], productive dust and fumes in the workplace [29], and air pollution [30]. Several studies conducted in Guangdong province showed that despite a high prevalence of cigarette smoking, a declining rate of cigarette smoking was observed in males in recent decades [31]. The lower rate of cigarette smoking (1.7%) observed in males accompanied a corresponding increase in the cigarette smoking rate in females [31, 32]. Thus, the rapid growth in the incidence of lung cancer in Sihui cannot be explained fully by cigarette smoking, especially in males.

It has been estimated that nearly half of deaths from lung cancer in China can be attributed to aging in China [33]. Based on the APC analysis, we showed that age cohort had a significantly positive association with the risk of lung cancer after adjusting for the period and birth cohort effects in both sexes, thus implying that increased longevity increases the risk of lung cancer in Sihui [34]. With the economic development in recent decades in Sihui, enhancements in public hygiene, improved nutrition, basic health care availability, and medical progress have brought an increase in life expectancy of its residents. In 2010, the average life expectancy in Sihui (76.6 years) was longer than the average life expectancy in China by 3.1 years [35].

In addition to the major risk factors (cigarette smoking and longer life expectancy), other known and suspected risk factors of lung cancer have changed considerably. For example, the rapid industrialization and economic development in the 1980s brought air pollution, dust, and fume contaminants to occupational environments, which is especially pertinent to males. After 20–30 years of effects, this may have resulted in the accelerated increase of lung cancer incidence in males from 2005 to 2010, which was demonstrated by joinpoint analysis (Fig. 2).

Birth cohort trends for lung cancer incidence usually suggest that the risk factors exposed to in early life have changed. The incubation time between exposure and cancer occurrence is 20–30 years for lung cancer [36], and lung cancer has the highest incidence in those ≥60 years of age, so the relevant age of exposure to risk factors would be in the first 4th decade of life. Thus, overexposure to lung carcinogens in Sihui should have occurred prior to the 1940s and 1950s and less frequently after the 1960s. We assume that the combination of a high prevalence of cigarette smoking, advanced age, and exposure to worsening air pollution in the younger generation might be the main reasons underlying the birth cohort effect on the risk of lung cancer in the last 25 years in Sihui.

Variation in the calendar period trend usually indicates the impact of new diagnoses, improved medical interventions, or a change in ascertaining or coding the cause of incidence. In this study, we observed a slight variation over the time periods studied. The slight increase in the calendar period trend coincided with changes in the incidence of lung cancer due to the use of the bronchofiberscopes in the 1990s, computed tomography in the 1990s [37], and tumor markers in the 2000s [38].

Our study had some limitations. Specifically, the histologic diagnostic information of patients was incomplete, so we could not analyze the incidence trends in lung cancer of different histologic subtypes. We also did not obtain the data of air quality and cigarette smoking in Sihui to analyze their relationships with the trends in lung cancer incidence.

In summary, a 3.4-fold increase in the incidence of lung cancer was noted in Sihui over the past 25 years. Age, period, and birth cohort effects on the risk of lung cancer in both sexes were observed, which suggests important roles for social aging, cigarette smoking, and environmental risk factors in the risk of lung cancer. Comprehensive measures including decreasing the prevalence of tobacco smoking in males, reducing the occupational exposure, and mitigating the air pollution may be taken to alleviate the burden of lung cancer in rural areas of South China.
